# Relative Preference and Localized Food Affect Predator Space Use and Consumption of Incidental Prey

**DOI:** 10.1371/journal.pone.0151483

**Published:** 2016-03-15

**Authors:** Tyler E. Schartel, Eric M. Schauber

**Affiliations:** 1 Cooperative Wildlife Research Laboratory and Department of Zoology, Southern Illinois University Carbondale, Carbondale, Illinois, United States of America; 2 Center for Ecology, Southern Illinois University Carbondale, Carbondale, Illinois, United States of America; 3 Department of Biological Sciences, Mississippi State University, Mississippi State, Mississippi, United States of America; University of Lleida, SPAIN

## Abstract

Abundant, localized foods can concentrate predators and their foraging efforts, thus altering both the spatial distribution of predation risk and predator preferences for prey that are encountered incidentally. However, few investigations have quantified the spatial scale over which localized foods affect predator foraging behavior and consumption of incidental prey. In spring 2010, we experimentally tested how point-source foods altered how generalist predators (white-footed mice, *Peromyscus leucopus*) utilized space and depredated two incidental prey items: almonds (*Prunus dulcis*; highly profitable) and maple seeds (*Acer saccharum*; less profitable). We estimated mouse population densities with trapping webs, quantified mouse consumption rates of these incidental prey items, and measured local mouse activity with track plates. We predicted that 1) mouse activity would be elevated near full feeders, but depressed at intermediate distances from the feeder, 2) consumption of both incidental prey would be high near feeders providing less-preferred food and, 3) consumption of incidental prey would be contingent on predator preference for prey relative to feeders providing more-preferred food. Mouse densities increased significantly from pre- to post-experiment. Mean mouse activity was unexpectedly greatest in control treatments, particularly <15 m from the control (empty) feeder. Feeders with highly preferred food (sunflower seeds) created localized refuges for incidental prey at intermediate distances (15 to 25m) from the feeder. Feeders with less-preferred food (corn) generated localized high risk for highly preferred almonds <10 m of the feeder. Our findings highlight the contingent but predictable effects of locally abundant food on risk experienced by incidental prey, which can be positive or negative depending on both spatial proximity and relative preference.

## Introduction

Predator foraging behaviors, and the resulting distribution of predation risk within a landscape, are influenced by what prey are available and where they are located. Optimal foraging theory provides a framework for predicting predator choice of prey on the basis of energetic profitability [1], as well as the spatial distribution of predator foraging efforts in relation to local prey availability [2, 3]. Increased abundance of generalist predators, which are numerically decoupled from the abundance of some prey, can increase the likelihood of localized extinction for sparse or rare prey items [4]. These sparse or rare items are especially vulnerable when encountered and consumed opportunistically as incidental prey while predators forage for primary, or locally abundant, foods [5]. Abundant food sources can supplement predator diets [6–9] elevate predator densities [10, 11], and influence generalist foraging strategies and space use [12, 13], suggesting that the distribution of primary food resources may play a crucial role in determining local risk to incidental prey.

Researchers have identified several scenarios whereby primary prey can influence the impacts of generalist predators on incidental prey. Primary prey sources can, when abundant, reduce predation risk for incidentally encountered and less-preferred prey items [14–19]. Alternatively, abundant primary prey can increase local predator densities through aggregative and numerical responses [20, 21], producing apparent competition that increases local predation rates on incidental prey that are preferred or highly vulnerable [22–24]. For example, deer feeders dispensing corn (*Zea mays*) spatially concentrate foraging by raccoons (*Procyon lotor*), increasing predation risk for nearby nests of wild turkeys (*Meleagris gallopova*) and turtles [25, 26]. On the other hand, locally abundant food also can draw predators away from opportunistically consumed prey items like waterfowl nests located in different areas [27].

These disparities in indirect effects of primary prey on incidental prey via a shared predator may be explained by the spatial scales at which predators are active and the preference ranking of available foods. However, few investigations have attempted to quantify the spatial scale at which spatially concentrated foods influence predator activity and foraging behavior. Optimally foraging generalists should preferentially consume the most profitable prey items available [28], so the relative profitability of abundant prey will determine (at least in part) whether incidental prey are consumed or disregarded. This reasoning raises a series of questions: (1) at what spatial scale do localized, abundant food sources influence predator space use and foraging behavior, (2) how do abundant food sources influence predator preference for other prey items, and (3) how does the profitability of abundant food influence consumption rates on incidental prey of differing profitability? To answer these questions, we provided abundant, localized food sources of differing profitability in order to manipulate predator space use and foraging behavior. We then quantified both predator activity and consumption rates on two incidental prey items of differing nutritional content relative to these localized food sources.

The white-footed mouse (*Peromyscus leucopus*) is an ideal predator for this investigation because of its generalist diet and small home range size (~0.1 ha; [29, 30]). Distributed widely across North America, the white-footed mouse consumes fruits and fungi [29, 31], and is an important predator of tree seeds [32–34]. White-footed mice are also noted predators of gypsy moth pupae (*Lymantria dispar*; [35–37]) and songbird eggs and fledglings [18, 38–40]. Abundant food sources may influence predation risk to white-footed mouse prey by concentrating mouse space use and altering mouse preference for prey items relative to what prey are available [34]. For example, highly preferred foods such as sunflower seeds (*Helianthus annuus*) may decrease predation on less-preferred incidental prey items (e.g., gypsy moth pupae; [41]). In addition, a locally abundant food source may draw mice away from areas farther away from this food source, generating refugia.

**Fig 1:**
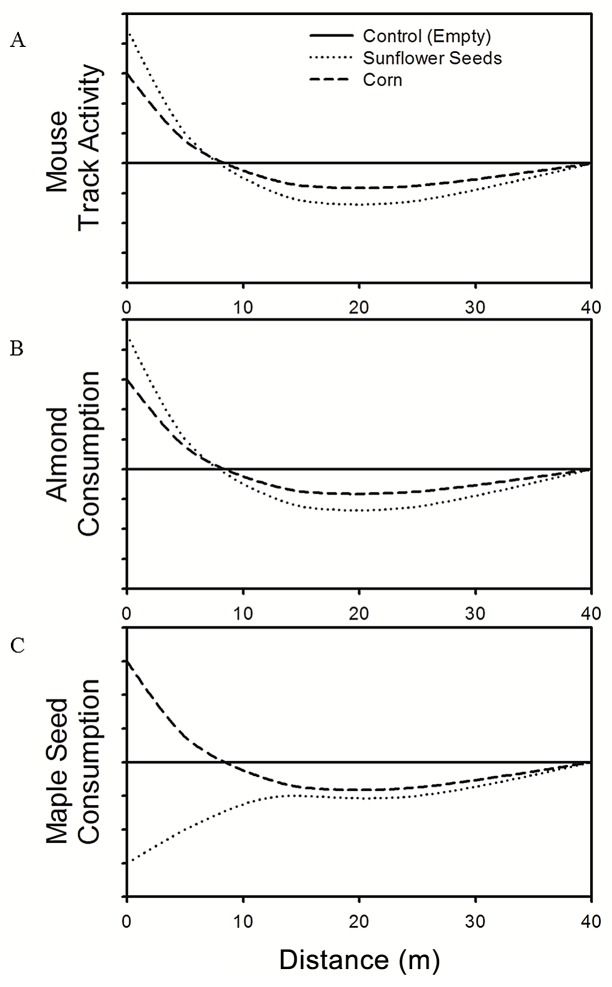
Predictions regarding the effects of abundant food on mouse space use, activity, and incidental prey consumption. (A) Predictions 1 and 2: Feeders provisioned with highly (sunflower seeds) and less-preferred (corn) food sources would concentrate mouse track activity near the feeder while simultaneously decreasing activity at intermediate distances (15 and 25m) from the feeder. (B) Predictions 3 and 4: Mouse consumption of highly preferred incidental prey (almonds) would be increased around (0 and 10m), and decreased at intermediate distances (15 and 25m) from, feeders provisioned with abundant, highly and less-preferred food. (C) Predictions 3 and 5: Mouse consumption of less-preferred incidental prey (maple seeds) would be increased around (0 and 10m), and decreased at intermediate distances (15 and 25m) from, feeders provisioned with abundant, but less-preferred food. Conversely, mouse consumption of maple seeds would be decreased around feeders provisioned with highly-preferred food, but would increase with distance from the feeder.

Our investigation aims to quantify and compare spatial patterns of white-footed mouse foraging behavior and activity with spatial patterns of predation risk to and consumption of incidental prey. In particular, we evaluate how localized and abundant, highly and less-preferred food resources affect and potentially generate discrepancies between patterns of mouse activity and consumption of incidental prey. We hypothesized that mice would forage for and consume incidental prey in a manner consistent with optimal foraging theory. It follows that we should expect the presence of a more-preferred food to cause mismatches in the spatial patterns of incidental prey consumption and mouse activity, especially near the food source. Given that white-footed mice are important predators of tree seeds, we used almonds (*Prunus dulcis*) and sugar maple seeds (*Acer saccharum*) as incidental prey. While not naturally occurring in this system, the nutritional profile of almonds (by weight; 24.9 kJ/g, 2.6% water, 22.1% protein, 52.8% lipids, 19.3% carbohydrates, [42]) suggest these seeds should be preferentially consumed relative to sugar maple seeds (by weight; 20.2 kJ/g). Sugar maple seeds have also been demonstrated to be consumed by white-footed mice with intermediate preference relative to other prey items [32, 34]. Abundant foods can cause consumers to become more selective, so alterations to mouse preferences for and consumption of incidental prey (maple seeds) may demonstrate the potential for effects on tree recruitment rates and plant diversity at scales consistent with mouse foraging [43]. In addition, these incidental prey items can be considered substitutes for other sessile prey of white-footed mice (e.g., gypsy moth pupae and songbird nests), so alterations to mouse preference for tree seeds may be indicative of similar potential effects on gypsy moth and songbird recruitment. We evaluate if abundant food sources influence local mouse densities, and thus mouse activity, space use, and foraging behavior. Finally, we discuss how heterogeneity in predation risk ultimately impacts the existence of refugia and the ability of incidental prey populations to exploit these areas of decreased risk. Specifically, we tested the following *a priori* predictions (Fig 1):

1. white-footed mouse space use and activity would be concentrated by supplemental food sources, especially around highly preferred food sources (sunflower seeds, Fig 1A).
2. white-footed mouse space use and activity would be decreased at intermediate distances from the feeder (15–25 m) due to activity being concentrated around the feeder (Fig 1A).
3. white-footed mouse consumption of all incidental prey would be high close to, and decrease with distance from, feeders providing less- preferred food (corn; Fig 1B and 1C).
4. white-footed mouse consumption of highly preferred prey (almonds) would be high close to, but decrease with distance from, feeders providing highly preferred food (sunflower seeds; Fig 1B).
5. white-footed mouse consumption of less-preferred prey (maple seeds) would be low close to, but increase with distance from, feeders providing highly preferred food (sunflower seeds; Fig 1C).

## Methods

### Study site and experimental plot design

This research had the approval of Southern Illinois University Carbondale's IACUC.The relevant animal care protocol # was 07–053. We conducted this investigation in spring 2010 at Southern Illinois University’s Touch of Nature Environmental Center, approximately 13 km south of Carbondale, Illinois, USA. Land surveys found dominant overstory species included white oak (*Quercus alba*), black oak (*Quercus velutina*), hickory (*Carya* spp.), and northern red oak (*Quercus rubra*; [44]). Noted prominent understory species including eastern redbud (*Cercis canadensis*), flowering dogwood (*Cornus florida*), and rusty black-haw (*Viburnum rufidulum*; [45]), however non-natives including wild rose (*Rosa multiflora*) and Japanese honeysuckle (*Lonicera japonica*) have invaded the forest interior [46].

We employed a 3-treatment (supplemental sunflower seeds, supplemental corn, or empty feeder control) crossover design with 6 plots as experimental units. Each plot contained concentric rings with radii of 5, 10, 15, 25 and 40 m centered on a feeder (0 m), as well as 8 radial trapping transects oriented along cardinal and secondary compass directions. All plots were spaced ≥ 100 m apart edge to edge, as well as ≥ 100 m from the forest edge. Each treatment was applied to a plot during a 2-week period, separated from other treatments by a 1-week recovery period. Small mammal population densities were estimated by live-trapping at the beginning and end of the experiment.

The feeder on each plot was constructed from a galvanized steel trash can (117 L) and lid. Four, 4-cm holes were drilled in the bottom of each can and 3.8-cm diameter polyvinyl chloride (PVC) tubes were inserted to minimize food loss and allow rodents to enter while excluding larger animals. Empty feeders served as control treatments, whereas *ad libidum* sunflower seeds and cracked corn were used as supplemental food sources (by weight: dried sunflower seed kernels: 24.4 kJ/g, 1.2% water, 19.3% protein, 49.8% lipids, 24.0% carbohydrates; cracked yellow corn: 15.3 kJ/g, 10.4% water, 9.4% protein, 4.7% lipids, 74.3% carbohydrates, [42]). We predicted that sunflower seeds would be highly preferred food and cracked corn would be less-preferred based on their respective energy densities and because *P*. *leucopus* appears to prefer foods that are energy-rich and approximately 15% protein (sunflower seeds, [47]). Each plot received a separate 2-week trial for each of the 3 food treatments (sunflower, corn, or empty) provided in the periods of 12–23 April, 3–14 May, and 24 May–4 June. The 6 possible food trial sequences (e.g., corn, empty, sunflower) were randomly assigned to the 6 plots. Food was removed at the end of each trial (i.e., feeder empty during the recovery period between trials) and the feeder was either refilled with another food treatment or left empty (control) at the start of the following period.

Abundant food sources can spatially concentrate predators, thereby increasing local predator densities and foraging efforts. We used trapping webs [48] to estimate pre- and post-experiment mouse densities. Paired Sherman live-traps (Model LFA; H. B. Sherman Traps, Inc., Tallahassee, Florida) were placed next to the feeder and along 8 trapping transects at each ring distance (5, 10, 15, 25, and 40 m), giving a total of 82 traps per plot. Each pair of traps was covered with a wood board to provide shelter against environmental conditions. Traps on all plots were baited with oats, provisioned with cotton bedding, and opened at ca. 1600 hr Sunday through Thursday in 2 consecutive weeks pre- (29 March–9 April) and post-experiment (7–18 June). Traps were checked and closed the following mornings at ca. 0800 hr. Each captured animal was marked with a Monel ear tag in each ear, examined to determine sex, reproductive condition, and age, then immediately released. Traps were not set or baited during the period when supplemental food or incidental prey were deployed.

### Quantifying rodent activity and consumption of incidental prey

Mouse activity was quantified using track plates, consisting of graphite-coated acetate sheets affixed to aluminum flashing [49, 50]. Rings at distances of 0, 5, 10, 15, 25 and 40 m received 4, 4, 8, 12, 20, and 32 track plates uniformly spaced, respectively, for a total of 80 track plates per plot. Track plates were monitored 4–5 times in each 2-week feeding trial at intervals of 1, 2, or 4 days (depending on day of plate deployment and accounting for weekends). All plates were closely inspected for the presence of tracks and, if present, tracks were identified to species. Tracked plates were marked to prevent double counting and replaced when tracks covered > 25% of the plate.

Our incidental prey items (almonds and sugar maple seeds) were prepared for field deployment by embedding them in unscented beeswax (Strahl and Pitsch Inc., West Babylon, New York, USA) on pieces of burlap [51]. This method of preparation required predators to expend some effort in consuming the prey items and to leave marks that could be used to identify the predator responsible for the depredation event. Burlap was cut into 4- x 4-cm squares and then double coated with beeswax. Short (1.3 cm) segments of 1.9-cm diameter PVC pipe, lightly coated with mineral oil, served as molds. A whole almond was placed inside a mold on a pre-waxed burlap square, and the mold was filled with molten beeswax until most of the almond was encased by wax. The wax was allowed to cool and the PVC mold was removed, leaving the almond affixed to the burlap. Maple seeds were affixed individually to burlap by spooning molten wax over the seed wing. All prey items were handled with latex gloves for the entirety of their preparation and deployment.

The schedule for incidental prey deployment was the same as that for food treatments and track plates. Rings at distances of 0, 5, 10, 15, 25, and 40 m received 4, 4, 8, 8, 12, and 12 of each incidental prey item, respectively, for a total of 96 prey items per plot. We deployed incidental prey items at random compass bearings within each ring, staking each into the ground using a bamboo skewer, and monitored them every 1, 2, or 4 days (same as for track plates) for each 2-week food trial. The presence or absence of each prey item was noted and, if depredated, the item was closely inspected for tooth-marks, pattern of damage, and the presence of scat. Consumption events were typically attributed to mice or raccoons based on tooth-marks or scat. Marks that were not distinctly mouse or raccoon were either discarded from future analyses or grouped together into an “unknown” predator category. If the item was present and intact, it was left in place. Each depredated item was replaced with a new prey item at a new random bearing within the same ring to avoid predators learning to return to sites of previous encounters.

### Data analysis

Live-trapping data from each trapping session and plot were analyzed using program DISTANCE to estimate mouse densities [48] before and after the experiment. We used program DISTANCE to evaluate a variety of detectability functions created using all possible combinations of key functions (half-normal, uniform, and hazard rate) and adjustment factors (cosine, simple polynomial, and hermite polynomial). Akaike’s Information Criterion for small samples (AIC_c_) was used to select the combination of key function and adjustment term which best balanced bias and variance, and to weight models for model-averaged density estimates [52]. We used a paired t-test to test whether model-averaged estimates of mouse density differed between pre- and post-experiment periods.

We conducted mixed-model logistic regression (PROC GLIMMIX; [53]) to test for main and interactive effects of treatment and distance on mouse activity (presence vs. absence of new mouse tracks on a plate during a check) and consumption of prey items (almonds or maple seeds; attacked vs. not attacked during a check), after accounting for period and varying intervals between checks. Plot was the experimental subject with random intercept, to account for non-independence of data from each plot, and food treatment, distance from the feeder, interval since the last check (1, 2, or 4 days), sampling period (first, second, or third), and the interaction of distance and food treatment were used as categorical explanatory variables. We tested for an interaction of distance and food treatment based on our predictions that different food treatments would produce different patterns of track activity or prey consumption at varying distances from the feeder. When this interaction was significant (α = 0.05), we used mixed-model logistic regression to test for a treatment effect separately for each distance from the feeder, again accounting for sampling period and the interval since the last check, and also to test for trends in track activity versus distance (continuous) from the feeder separately for each food treatment after accounting for interval since last check (some analyses including period failed to converge). We also analyzed consumption rates separately for mouse-only and mouse+unknown predator groups. All raw data pertaining to small mammal trapping, predator activity and space use, and consumption of incidental prey items have been deposited at Dryad, DOI: 10.5061/dryad.f8rm5

## Results

We captured 166 mice a total of 483 times over 10 332 trap nights. Our trapping web data lent the most support to a half-normal, cosine detectability function from both pre- and post-experiment trapping sessions, but considerable support remained for 2 alternative functions in each period (Table 1). Model-averaged estimates of pre-experiment densities ranged from 1.9 to 4.0 mice/ha (mean = 3.05 mice/ha) and increased 123% to 216% (Paired *t* test: t5 = -3.04, *P* = 0.014) to post-experiment estimates of 3.8 to 10.3 mice/ha (mean = 5.65 mice/ha; Table 1).

**Table 1:** Pre- and post-experiment population density estimates for white-footed mice. Density estimates were generated from program DISTANCE for pre- and post-food treatment trapping sessions on 6 experimental trapping webs located at Touch of Nature Environmental Center, Carbondale, Illinois, USA. Detectability functions were created using half-normal (HN), uniform (UN), and hazard rate (HR) key functions combined with cosine (COS), simple polynomial (SP), and hermite polynomial (HP) adjustment factors.

					Estimated Density (mice / ha)					
Period	Model	k^*^		AIC_c_	Plot 1	Plot 2	Plot 3	Plot 4	Plot 5	Plot 6
Pre	HN+COS	9		0.54	3.41	5.08	3.31	2.23	1.89	4.31
	HN+HP	6		0.22	1.70	2.57	3.31	2.23	1.89	2.56
	HN+SP	6		0.22	1.70	2.57	3.31	2.23	1.89	2.56
	HR+COS	12		0.01	6.58	6.30	2.34	4.19	1.82	6.32
	HR+SP	13		0.01	6.58	6.30	45.38	3.82	1.82	6.32
	HR+HP	13		0.01	6.58	6.30	35.18	3.82	1.82	6.32
	UN+COS	11		0	1.35	4.49	2.9	1.79	1.46	3.58
	UN+SP	6		0	1.14	1.7	1.94	1.53	1.27	1.4
	UN+HP	11		0	0.92	1.94	1.8	1.76	1.43	1.46
	**Model average**				**2.73**	**4.01**	**3.79**	**2.26**	**1.89**	**3.59**
Post	HN+COS	12		0.39	4.85	4.30	2.40	3.57	4.00	3.98
	HR+COS	12		0.29	6.82	5.72	16.21	5.21	3.73	4.84
	HR+SP	12		0.29	6.82	5.72	16.21	5.21	3.73	4.84
	HN+SP	6		0.01	2.79	2.39	1.24	1.9	2.35	1.86
	HN+HP	6		0.01	2.79	2.39	1.24	1.9	2.35	1.86
	HR+HP	6		0.01	2.79	2.39	1.24	1.9	2.35	1.86
	UN+COS	15		0	4.7	2.53	1.14	3.33	3.42	2.91
	UN+SP	6		0	1.65	1.57	0.95	1.28	1.33	1.02
	**Model average**				**5.91**	**5.05**	**10.31**	**4.46**	**3.78**	**4.4**

^*^ k indicates the number of parameters in each model.

**Fig 2:**
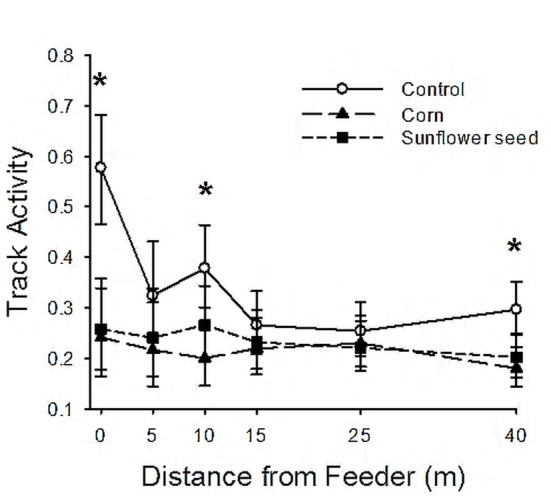
Estimated mouse track activity (tracked plates per plate-check) by distance from feeder in 3 food treatments. All 6 experimental plots were located at Touch of Nature Environmental Center, Carbondale, Illinois, USA in spring 2010. Estimates are based on least-squares means from generalized linear mixed model, accounting for experimental period and varying intervals between plate checks. Error bars are 95% confidence intervals. Significant differences among treatments are indicated by asterisks.

**Fig 3:**
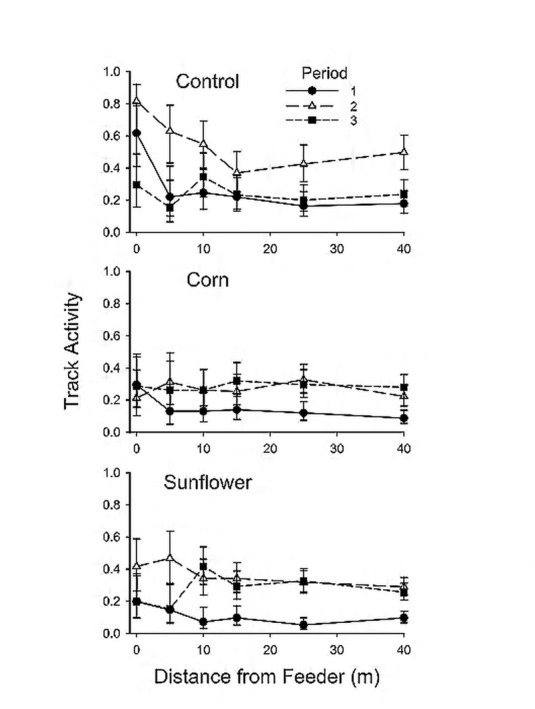
Period-specific, estimated mouse track activity (tracked plates per plate-check) by distance from the feeder. Food treatments (A) control (empty feeder), (B) corn, and (C) sunflower seeds were provided on 6 experimental plots located at Touch of Nature Environmental Center, Carbondale, Illinois, USA in spring 2010. Estimates are based on least-squares means from generalized linear mixed model, accounting for varying intervals between plate checks. Error bars are 95% confidence intervals.

We found significant interactive effects of treatment and distance on mouse track activity (Table 2), as expected, but activity patterns deviated from our first two predictions. We had predicted that track activity would increase near the feeder when food was provided (Fig 1A), but mean track activity was greatest near the feeder in control (empty) treatments (Fig 2). Track activity declined with distance in each treatment separately, but more strongly in control (β ± SE = -0.012 ± 0.0034) than in corn (-0.0076 ± 0.0037) or sunflower (-0.0085 ± 0.0037) treatments. Elevated mouse activity near control feeders could result if mice had learned to associate the feeder with food or if the feeder still smelled of food, but mouse activity was actually greatest near the feeder in plots where the control treatment occurred first—i.e., when the feeder was clean and empty (Fig 3). In contrast, mouse activity around control feeders in period 3 was comparable to activity observed around corn/sunflower treatments in the same experimental period. Thus, providing food reduced the degree to which activity near the feeder was elevated relative to control treatments, at least early in the experiment.

**Table 2:** Results of mixed-model logistic regression analysis of the frequency of plates tracked vs. study parameters. Analysis was conducted on track plate data collected on 6 plots located at Touch of Nature Environmental Center, Carbondale, IL, USA in spring 2010.

	df			
Parameter	num	denom	F	P
Treatment	2	10	31.35	<0.0001
Distance	5	25	5.83	0.0011
Interval	2	10	62.48	<0.0001
Period	2	10	136.89	<0.0001
Treatment*Distance	10	50	3.01	0.0047

A total of 2971 almonds and 1527 maple seeds were attacked by predators. For almonds, 39% of attacks were attributable to mice, 6% to raccoons, and 54% unknown. Only 4 almond consumption events were attributed to predators other than mice or raccoons. These 4 events were removed from future analyses. Few attacks on maple seeds were confidently attributed to either mice (5%) or raccoons (1%), so the remainder of attacks (94%) were grouped together and attributed to unknown predators. As a result of small sample size, analysis of mouse-only consumption of maple seeds failed to converge.

**Fig 4:**
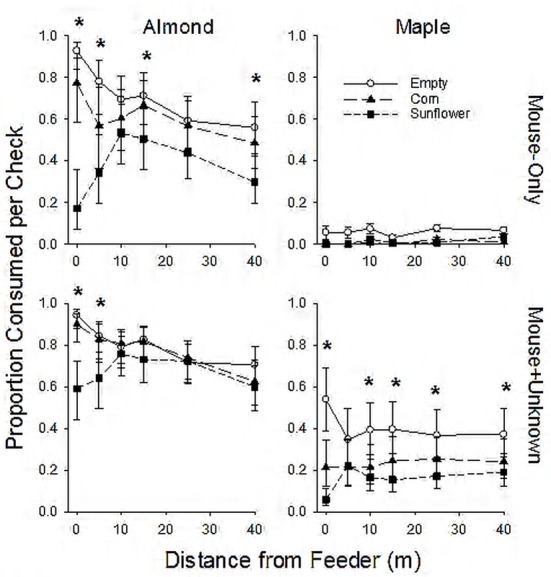
Mean (±SE) consumption of incidental prey by mouse-only (PL only) and mouse and unspecified predators (PL + unknown). Consumption of incidental prey was recorded as the proportion consumed per check (1–4 days) during 3 food treatment (control (empty), corn, and sunflower) periods on 6 experimental plots located at Touch of Nature Environmental Center, Carbondale, IL, USA in spring 2010. Estimates are based on least-squares means from generalized linear mixed model, accounting for experimental period and varying intervals between plate checks. Error bars are 95% confidence intervals. Significant differences among treatments are indicated by asterisks.

Overall mean consumption rates of both almonds and maple seeds were greatest in control treatments and tended to be lowest in sunflower treatments (Fig 4). Consumption rates also generally increased from period 1 to period 3 (Table 3). We found significant distance×treatment interactions for mouse-only almond consumption and maple seed consumption by mouse+unknown predators, but not for almond consumption by mouse+unknown predators (Table 3). Consumption of both almonds and maple seeds was reduced near feeders filled with sunflower seeds (Fig 4). Quantitatively, however, the reduction in consumption rates was much greater for maple seeds: estimated consumption rates by mouse+unknown predators (based on least-squares means to correct for period and interval since last check) in the immediate vicinity of feeders with sunflower seeds were 6% for maple seed versus 59% for almonds (Fig 4).

**Table 3:** Results of mixed-model logistic regression on consumption of almonds and maple seeds by predator group (mouse-only and mouse+unknown predators). Analysis was conducted on data concerning predator consumption of incidental prey items. These data were collected on 6 experimental plots located at Touch of Nature Environmental Center, Carbondale, IL, USA in spring 2010. Results of mouse-only predation on maple seeds are not presented as convergence was not reached during analysis.

			df			
Prey item	Predator group	Parameter	num	den	F	P
Almonds	Mouse-Only	Treatment	2	10	46.03	<0.0001
		Distance	5	25	6.95	0.013
		Interval	2	10	107.66	<0.0001
		Period	2	10	213.71	<0.0001
		Treatment*Distance	10	50	3.62	0.0013
Almonds	Mouse+Unknown	Treatment	2	10	24.48	0.0001
		Distance	5	25	13.44	<0.0001
		Interval	2	10	118.22	<0.0001
		Period	2	10	324.87	<0.0001
		Treatment*Distance	10	50	4.23	0.0003
Maple seeds	Mouse+Unknown	Treatment	2	10	68.09	<0.0001
		Distance	5	25	0.59	0.71
		Interval	2	10	115.66	<0.0001
		Period	2	10	313.88	<0.0001
		Treatment*Distance	10	50	2.96	0.0053

## Discussion

The distribution and abundance of food can influence predator space use and foraging efforts [12, 13], indirectly influencing risk to other prey. Predators can become spatially concentrated by abundant localized food sources, and the consequences of such "hot spots" of predator activity for incidental prey differ depending on preference ranking: elevated risk to highly preferred incidental prey but a zone of safety for less-preferred prey [54–56]. Within this context, we sought to compare spatial patterns of mouse activity and consumption of incidental prey items. We evaluated these spatial patterns, and discrepancies between them, relative to the presence or absence of supplemental foods. On this basis, we predicted that providing abundant localized food would 1) concentrate mouse space use near the feeder and 2) potentially depress mouse activity at intermediate distances (out to the diameter of a mouse home range). Instead, track activity was slightly elevated close to the feeder (< 15 m) in both food treatments, but was strongly (and unexpectedly) elevated near control (empty) feeders. This elevated mouse activity around empty, control feeders is most consistent with the initial novelty of the feeder, as the relative response was strongest during the first experimental period when clean feeders were provided in control treatments and mice had no prior experience with them. Alternatively, elevated activity around control feeders may reflect mice seeking shelter or frustrated foraging attempts by mice that had learned in previous experimental periods to associate the metal cans with food, but these hypotheses are countered by the empty feeder effect being weakest in the third experimental period.

We did observe elevated mouse activity near the filled feeders, which represents a combined effect of both the feeder itself and the provided food. Relative to control treatments, the presence of food in the feeder appeared to reduce the degree to which activity near the feeder was elevated, which was unexpected. We see two potential explanations for this unexpected effect of food: either corn and sunflower seeds reduced the attractiveness of feeders (relative to empty feeders) or it altered the behavior of mice in the vicinity of the feeder. White-footed mice are largely granivorous, so the first explanation merits little consideration. And, filled feeders had the same design to exclude larger animals as empty ones, so they provided shelter as well. Instead, we argue the net reduction in local mouse activity around filled feeders (relative to controls) likely indicates that mice responded to abundant food by shifting their foraging from the area around the feeder to inside the feeder itself. Elevated food availability can reduce foraging activity in general and can reduce the amount of space that is intensively foraged by increasing giving up densities [5, 57–59]. The presence of reliable food in a specific location would be expected to particularly decrease the benefits of foraging elsewhere.

Regarding our second prediction, we did not observe a general pattern of depressed track activity at intermediate distances from either filled or empty feeders. This lack of result is likely related to the difference in spatial areas: for example, the annulus between radii of 10 and 25 m has an area 21 times larger than the circular area within 5 m of the feeder. Thus, the shift of a fixed amount of mouse activity from the annulus to the circle would cause an increase in activity density near the center 21 times greater than the corresponding decrease in the annulus. Such a subtle decrease in activity is likely to require very high sample size to reliably detect. However, even subtle modifications in predator activity, and thus predation risk, over large areas can disproportionately increase prey survival rates. Concentrating predation risk in space generally increases overall survival of relatively sessile prey because depletion of prey in high-risk areas quickly generates a negative spatial correlation between prey abundance and risk [60, 61]. Jensen's inequality [62] further amplifies the influence of a modest reduction in risk over a large area, because probability of survival over an extended period is a convex (exponential) function of daily survival rates. For example, if daily survival averages 0.9 but varies among individuals (0.6 for one individual, 0.93 for 10 others), overall survival over 10 days exceeds the value expected from the average daily rate: 0.9^10^ = 0.35 versus [0.6^10^ + 10*0.93^10^]/11 = 0.44. This subtle but potentially important reduction in risk is an important consideration when interpreting results showing elevated risk of generalist predation near artificial food sources [56, 63, 64].

Regardless of the cause of spatial variations in mouse activity, we expected that consumption rates of incidental prey would reflect the local risk of discovery, as indicated by mouse activity, mediated by predator preference for these prey relative to the provided food treatment. Based on these expectations, our third prediction was that consumption rates for both incidental prey items would be elevated near feeders providing a less-preferred food (corn). This prediction was partially supported for almonds but not for maple seeds. On the other hand, sunflower seeds have higher energy density and protein content than corn, so we predicted that feeders providing sunflower seeds would elevate local consumption of almonds but depress consumption of less-preferred maple seeds. However, consumption of both almonds and maple seeds was depressed near feeders providing sunflower seeds. Taken together, these deviations from our predictions regarding consumption rates imply that the incidental prey items we deployed were consistently devalued relative to the food provided in feeders. Incidental prey may be devalued by handling time cost imposed by seeds being embedded in wax. However, predation by small mammals (mainly white-footed mice) was much greater for freeze-dried gypsy moth pupae affixed to burlap with beeswax than for naturally occurring gypsy moth pupae at the same microsites and times [65], so wax and burlap do not appear to deter these predators. A more promising explanation is that mice found both food and safety within our feeders, such that food items deployed in the open incurred foraging costs due to risks of attack by the rodents' own predators [66]. We also suggest that our results regarding consumption rates on maple seeds should be interpreted with caution. Identifying maple seed predators was impeded because identifying marks (tooth-marks and scat) were rare—this problem stemmed from embedding the seed wing rather than the whole seed in wax. As a result, low sample size of the mouse-only predation events prevented model convergence. Given that confirmed mouse attacks made up 42% of mouse+unknown attacks on deployed almonds, we argue that patterns in attacks by the mouse+unknown predator group likely are driven by patterns in risk of attack by mice. Our results appear to show that predation risk to almonds increased more in proximity to feeders providing corn than track activity did, whereas we had predicted that predation risk to highly preferred prey should show a similar spatial pattern of track activity. This difference was not great, given the imprecision of the estimates, but if this mismatch is real it suggests our track plates may not have faithfully represented mouse activity in food treatments. Mice provisioned with abundant food sources may have spent more time foraging in a directed manner (i.e., right at the feeder) rather than searching across larger spatial scales [39, 67]. Consequently, track activity may have underestimated actual foraging intensity near abundant food sources. This highly directed foraging activity near the feeder, and decreased time spent foraging for other prey, could manifest as increased dietary selectivity for the provided food and decreased consumption rates of incidental prey.

The findings of this study may be applied to other generalist consumers and their prey (seeds and animals). Abundant food sources can decrease rodent predator activity levels [57, 58], influence site selection [5, 59] and result in less uniform distributions across small-scale habitats [31]. However, the spatial scale of this mechanism is poorly understood. We found that abundant food slightly elevated mouse space use and activity at distances ≤ 10 m, and in turn, predation risk to incidental prey at these distances. The concentrative effect of abundant food was less than predicted, indicating that providing food may not generate refugia for prey by displacing mouse activity and decreasing consumption rates on incidental prey away from the feeder. In general, rodent diet selection and space use can be influenced by the abundance and profitability of food sources; fox squirrels (*Sciurus niger*) over-utilized poor-quality habitat patches [68] and decreased diet selectivity when the abundance of food sources was increased [69]. Differences in incidental prey consumption between food treatments indicated that the palatability and profitability of an incidental prey item relative to that of an abundant food source can influence incidental prey consumption. In addition, the distance of the incidental prey item from the food source may contribute to determining whether incidental prey are consumed or disregarded, especially if these prey items are located near the food source. Conversely, removal or depletion of food sources may force predators to increase the rate and spatial scale of their foraging efforts, thereby potentially decreasing encounter and consumption rates on incidental prey. However, we found evidence that the absence of food resulted in higher mean activity levels and that this increased activity coincided with increased consumption of both incidental prey items, including less-profitable maple seeds.

The results of this investigation may be broadly applied to predator and prey interactions. Optimal foraging theory provides a general predictive framework for predator foraging behavior and choice of prey. However, generalist predators can deviate from optimal expectations by altering their space use and consuming suboptimal prey. Large predatory mammals, like African lions (*Panthera leo*), deviated from optimal foraging expectations by choosing suboptimal prey based on prey group size, prey distance from the hunting group, and prey group composition [70]. Avian consumers, when concentrated by bird feeders, became more selective and increased localized predation on incidental prey [56]. Differential space use and consumption of prey by predators suggests practical management implications for invasive, endangered, and game species. Our results suggest that providing abundant food sources near areas of high pest densities may encourage predators to aggregate and increase consumption rates on these incidental prey, provided the pest species is more profitable than the provided food.
